# Supplementary material for: Maps of Constitutive-Heterochromatin Distribution for Four Martes Species (Mustelidae, Carnivora, Mammalia) Show the Formative Role of Macrosatellite Repeats in Interspecific Variation of Chromosome Structure
Source: Genes (Basel). 2023 Feb 14;14(2):489. doi: 10.3390/genes14020489 (PMC9957230; doi:10.3390/genes14020489)
Supplement: Supplementary file 1 [file genes-14-00489-s001.zip › Figure S1. Negative FISH on cat chromosomes.pdf]

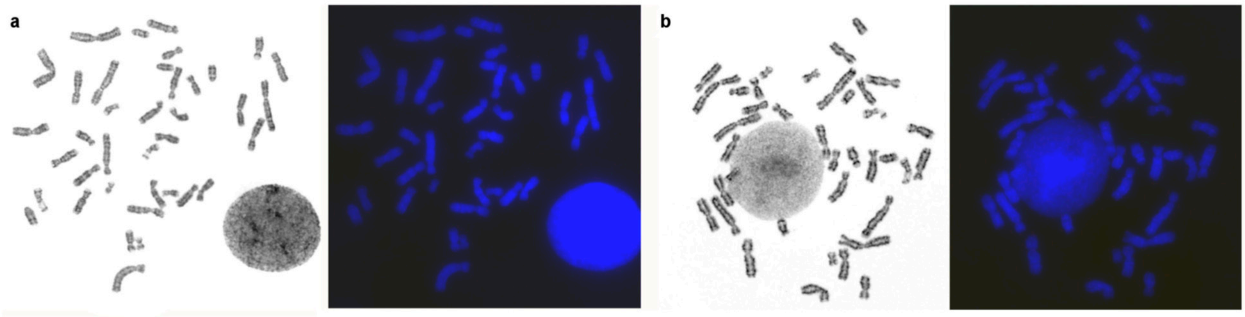

Figure S1. Examples of negative result of FISH with (a) MSRs S22+S26 probes and(b) MSRs S40+S46 on the domestic cat chromosomes.
